# Infection dynamics: from organ to host population

**DOI:** 10.1098/rsif.2007.1055

**Published:** 2007-04-17

**Authors:** T.J McKinley, James Wood

**Affiliations:** Department of Veterinary Medicine, Cambridge Infectious Diseases ConsortiumMadingley Road, Cambridge CB3 0ES, UK

**Keywords:** mathematical modelling, within-host spread, population spread, infectious animal diseases

## Abstract

A symposium discussing collaborative research work on infectious diseases dynamics was held at Queens' College, University of Cambridge on 25 October 2006.

The evolution and spread of infectious diseases are determined by dynamic processes occurring at many scales from the within-cell to the host population level. There is a considerable similarity between these different scales, whether considering the colonization and spread of salmonella within a cell or organ (Brown *et al*. 2006), the within-host dynamics of influenza, or the spread of directly transmitted infections such as rabies in wildlife (Russell *et al*. 2005), foot-and-mouth disease in livestock (Keeling *et al*. 2001) or rhizomania through sugar beet (Stacey *et al*. 2004). Better understanding of the underlying dynamics of these epidemiological processes is of paramount importance in the control and the prevention of infectious disease spread; the combination of mathematics with research into fundamental biological mechanisms can provide considerable insight into disease pathogenesis spread and control. This scenario provided a focus for a meeting on 25 October 2006 at Queens' College, Cambridge, organized by the Cambridge Infectious Diseases Consortium (CIDC).

CIDC was established to enhance veterinary education and research in infectious diseases, following the recommendations of the Royal Society's enquiry into ‘Infectious Diseases of Livestock’ (Follett *et al*. 2002) that the government needed to increase funding in this area. The CIDC involves collaborations between various departments in the University of Cambridge and several national research institutions in the area. The central focus of the training, education and research programmes of CIDC is the study of infectious disease dynamics; with particular emphasis placed on ensuring effective collaborations between virologists, microbiologists, epidemiologists and mathematicians. This philosophy was reflected by the day's programme, which showcased cross-disciplinary work being carried out on a range of different diseases and infective processes.

The meeting opened with a presentation from Dr Clare Bryant, a cell biologist from Cambridge, describing results from her collaborations with Dr Julia Gog (Department of Applied Mathematics and Theoretical Physics, University of Cambridge) into the *in vitro* dynamics of salmonella–macrophage interactions. The cross-disciplinary collaboration had resulted in new hypotheses and approaches in terms of experimental design. This was illustrated by some recent macrophage infection studies, performed by Ph.D. student Alicia Murcia, in which a number of initial assumptions were made based on widely accepted biological theory. These include treating the macrophage cell population as equally susceptible to infection, assuming that salmonella will kill infected macrophages in order to spread to other macrophages and salmonellae proliferate within macrophages. Following careful experimental studies to test a simple mathematical model based on these widely accepted assumptions, it is clear that not all macrophages are susceptible to infection; macrophage cell death may not be a pre-requisite to bacterial spread and although intracellular bacterial growth occurs, this cannot account for the total increase in intracellular bacteria. Hence, the question arises: are salmonellae able to re-infect infected macrophages? In the broader context, the mathematical–biological collaboration has resulted in a clearer view of the types of questions that need to be addressed experimentally and helped to refine the number and the type of experiments that need to be performed.

The session then swapped focus from salmonella to *Campylobacter* infections when Dr Chris Coward (Department of Veterinary Medicine, University of Cambridge) presented some work investigating the dynamics of the disease at the within-host level in chickens, based on findings from some previous *in vitro* studies of *Campylobacter* replication.

*Campylobacter* species are a major form of human food poisoning, with *Campylobacter jejuni* the leading cause of bacterial gastroenteritis in humans, responsible for over 40 000 laboratory-confirmed cases a year in England and Wales (http://www.hpa.org.uk/infections/topics_az/campy/data_ew.htm). A major source of human infection is thought to be consumption of contaminated poultry, there being a major reservoir of infection in the poultry industry where the organism behaves as an enteric commensal. Understanding how the numbers of organisms in poultry populations can be reduced provides a strategy for dealing with the problem in humans.

Previous research at Cambridge had focused on the identification of bacterial genetic factors important for the colonization of chickens using the technique of Signature Tagged Mutagenesis (STM; Holden & Hensel 1998). In STM, pools of individually identifiable mutants are screened through a model, in this case for colonization of the gastrointestinal tract of chickens to identify colonization-defective mutants. The work showed that colonization-proficient mutants were lost during mixed infections in an unpredictable way and led to hypotheses that bottlenecks in establishing colonization, between-bird transmission and phenotypic (phase) variation were responsible for this phenomenon (Grant *et al*. 2005). Early results were presented which suggested that bottlenecks of transmission were important features of population dynamics within hosts but were hard to predict.

Dr John McCauley from the National Institute for Medical Research at Mill Hill followed this with a presentation describing some of the different mechanisms that underlie the variation in host tropism of different influenza viruses, addressing some of the mechanisms other than just virus binding which might be responsible for both the failure and the success of H5N1 viruses to invade the human population and respiratory tract. Interferon (IFN) responses are important components of host cells' responses to virus challenge. Having described the qualitative differences between different virus approaches to evading a cell's interferon defences, he went on to describe a series of experiments relating to how different NS1 proteins in influenza viruses have their effects. Using a combination of different wild-type virus, as well as several others constructed using reverse genetics techniques, Dr McCauley described the different levels (i) of interferon induced by different human viruses, (ii) of signals induced by different influenza NS1's, and (iii) of sensitivity of different human viruses to the effects of IFN. Interestingly, the human viruses that induced most IFN were the least sensitive to its effects. Finally, experiments were described in which replication of highly pathogenic avian influenza viruses could be markedly enhanced by IFN interfering co-infections or disabling the interferon response of cells. This raised the question of whether more recent viruses, such as the recent H5N1 viruses, had already found a means of subverting this response and whether we should be measuring the host innate immune response when considering the ability of a virus to infect different species.

The final talk of the morning session was given by Dave Balkissoon, a CIDC supported student based at the BBSRC Institute for Animal Health, who presented results from investigations into the innate immune response to avian influenza in inbred lines of chickens. Another major public health issue, the potential socio-economic effects of avian influenza have been highlighted recently with mass media attention focused on the outbreak of the H5N1 strain of the disease currently moving across Asia, Africa and Europe. The poultry industry in the UK deals with approximately 40 billion chickens per year, including broilers, layers and breeders, as well as feral and wild birds, and the disease is highly contagious within avian populations. In addition, it has a high epizootic potential, and has so far (December 2006) killed 157 people worldwide (http://www.who.int/csr/disease/avian_influenza/country/cases_table_2006_12_27/en/index.html).

Of particular interest in this project is the role of the *Mx* gene in conferring resistance to infection from the influenza virus. These have been shown to affect anti-viral activity against some RNA viruses in various species (e.g. rats, mice, dogs and humans), but their role as an anti-viral agent specifically with regards to influenza in chickens is unclear. Additional interest lies in determining whether a relationship exists between the *Mx* gene and particular production traits, synonymous with the intensive selection processes used in commercial poultry lines. This can help to determine the levels of risk among commercial poultry stocks.

Initial results suggested a very heterogeneous distribution of the *Mx* anti-viral allele between different commercial and experimental poultry populations. Various production traits have been identified close to the *Mx* gene on chromosome 1 such as thigh muscle yield, tarsometatarsal length and leg twisting score, and whether differences are due to co-selection or chance founder effects was being investigated.

The afternoon session saw the scientific focus change from the biological aspects of infectious diseases highlighted in the morning, to the role that mathematical modelling can play, in both understanding the pathogenesis of diseases at a molecular level and also in aiding investigations into the dynamics of epidemic processes at the population level.

The opening talk was given by Dr Olivier Restif, a mathematical modeller working in CIDC, who examined the effect of widespread vaccination policies on the potential re-emergence of diseases due to vaccine-induced antigenic variation. The notion that certain control strategies can have an effect on epidemic development opposite to that intended is a constant concern for those implementing policy in public health. However, there are various recent examples of situations in which countries with high vaccination coverage from certain diseases have experienced increasing number of cases of the disease from new strains of the antigen. The modelling strategy investigated the effects of cross-immunity (i.e. the reduction in susceptibility caused by infection from a different strain) on the invasion and persistence of a novel strain of a disease to a finite population where an original strain of the disease is endemic. A series of stochastic simulations were used to assess the impact of cross-immunity when different levels of vaccination coverage were used.

The results from a series of stochastic simulations indicate that there is an important trade-off between invasion and persistence of the novel strain, with high levels of cross-immunity resulting in an increased probability of the invading strain of the disease replacing the existing one. In the presence of vaccination, the growth rate of the novel strain depends on the coverage of the vaccine and the level of cross-protection (over and above natural cross-immunity) conferred by the vaccine. Based on invasion analysis only, the worst case scenario is expected in a situation where there is high coverage of a vaccine that confers little cross-protection to the novel strain. However, stochastic simulations show that epidemiological feedback can prevent the persistence of highly invasive strains in a finite-size population. As a result, the model predicts that the most successful strains (i.e. which can invade and persist) can be either very close to or very far from the vaccine strain in terms of antigenic distances (Restif & Grenfell 2007). Accounting for these sorts of issues is the key in developing efficient reaction and eradication policies for endemic infections. The importance of adapting strategies in the face of increased knowledge is vital in preventing large-scale epidemics and this key issue was also revisited later in the day.

Beforehand there was a very different and novel application of mathematical techniques to help quantify antigenic variation, this time between rabies virus isolates. Rabies is a lyssavirus from the Rhabdoviridae family. It is a highly contagious epidemic disease that is almost invariably fatal. The standard control procedure used by the World Health Organization is vaccination, although the potential of the virus to mutate over time makes designing and implementing effective and efficient vaccination strategies difficult.

Understanding the antigenic variation in lyssaviruses could help to provide important insights into the evolution of the rabies disease and the dynamics of its spread, aiding selection of vaccine strains and prediction and control of its spread. Daniel Horton, another CIDC student, this time based at the international rabies reference laboratory at VLA, presented some work on using antigenic cartography (Smith *et al*. 2004) to make a visual map of the antigenic variation among lyssaviruses. This allows a quantitative measure of the differences between virus isolates to be obtained, and is potentially an extremely useful epidemiological tool, having been tested extensively on mapping influenza. However, knowledge of antigenic variation in lyssaviruses is poorly understood, and the focus of the work in this area is to produce robust antigenic maps of the rabies virus isolates and to extend current knowledge of recombinant lyssaviruses.

Initial findings show good associations regarding the expected genetic relationships of the isolates as well as some interesting differences in pathogenicity and incubation periods between different isolates of the same genotype (GT1). The maps have also proven to be experimentally robust.

Moving on from the application of mathematical modelling at the molecular level to its use at a somewhat larger scale, the next presentation was the first of two concerned with modelling the spatial spread of infectious disease spread across landscapes. Dr Colin Russell (Department of Zoology, University of Cambridge) continued the rabies theme and presented results from a stochastic mathematical model for the spatio-temporal spread of the disease in raccoons in northeastern USA (Russell *et al*. 2005).

Rabies causes noticeable behavioural changes in infected animals. Principal examples of this are increased aggressiveness, an increase in aberrant roaming patterns and extreme hydrophobia. The disease is spread through direct contact and the former two characteristics exacerbate the spatial progress of the disease into new areas; however, the latter feature can also have a direct impact on the course of an epidemic due to the effect of large bodies of water either blocking or funnelling the path of the infection.

The mathematical model incorporated spatial heterogeneity caused by water features such as rivers or lakes into the model, and used it to predict the spatial expansion of a historical epidemic in New York State, using data collected from Connecticut to parameterize the model. The predictive path of the disease corresponded well to the observed epidemic, even though the Connecticut and New York datasets were collected at different scales. It was found that there was a major slowing effect in the rate of local transmission of the disease when townships were separated by major rivers. An intriguing question then arises as to whether this knowledge could be used to guide and influence more effective control policies by incorporating water sources directly as part of a control method—possibly in conjunction with vaccination corridors.

The model was then used to predict incidence of the disease in Ohio, which at the current time was rabies free, principally due to a large vaccination corridor existing along the Ohio–Pennsylvania border. In a rather perverse twist of fate, soon after the predictions were obtained rabies did indeed break through the vaccination barrier and enter Ohio. As a consequence, the results of the study were used to help advise on a series of control policies, and so far the outbreak has been kept in check. Another theoretical result of the study suggests that combining vaccine barriers and rivers is a useful containment strategy. However, correct positioning is important with the optimal solution for controlling spread being to place the vaccine barrier on the opposite side of a large body of water to the approaching epidemic wavefront.

In contrast to the animal work that had come before it, Prof. Chris Gilligan (Department of Plant Sciences, University of Cambridge) focused instead on modelling the spatial spread of infectious diseases in plants. The previous discussion highlighted the importance of accounting for landscape heterogeneity when modelling epidemics, and due to factors such as population expansion, climate change and changes in farming practices, landscapes are changing dramatically, having a major effect on the extent of epidemic progression. An important point given was that the dynamics of the disease vary over different spatial scales—at the plant, within-field and farm premise levels—and these differences need to be reflected in both the modelling strategy and the control mechanism.

These ideas were illustrated with various examples of current work within the Gilligan group, most notably in some investigative work on the effects of different scale control policies on the propagation of rhizomania in sugar beet. This is a particularly persistent disease caused by a virus transmitted through the soil and into the roots by a carrier vector. It causes significant problems with regards to yield and is generally spread over large distances through the movement of agricultural equipment that can carry the virus from infested soil.

The traditional response strategy is by a local containment policy, involving removal of the infected crop, prevention of future growth in infested fields and the disinfection of agricultural equipment. A key focus in planning many control policies (as seen in the previous talk) is in trying to optimize the level of protection needed to prevent epidemic situations. Some interesting questions arise: for example, is there a minimum effective control neighbourhood, and should preference be given to protecting the more, or less infected regions? The idea that more intuitive control policies are sometimes much less effective than less intuitive ones echoes the results from the earlier presentation by Dr Restif. Some modelling simulations of the spread of disease in a host meta-population suggested that protection of the less infected regions proved more efficacious in controlling disease spread than protection within the infected regions. Additionally, not all sites needed to be protected to prevent an epidemic developing. It was shown that of greater importance is ensuring that the scale of the control policy matches the scale of the epidemic; in the rhizomania model for example, farm level control policies proved a much more effective preventive measure than field level policies.

Economic and logistical constraints are also the keys in instigating response strategies, and some additional work was presented linking epidemiological and economic models in order to optimize resources in epidemic situations. This fittingly led to the final presentation of the day, given by Dr Cathy Roth of the Global Outbreak Alert and Response Network (GOARN)— part of the World Health Organization (WHO)—in which she discussed the many problems facing policy makers when developing and implementing response strategies in the face of potential disease outbreaks. This drew together aspects from all the research work presented throughout the day, from the molecular scale right to population level, and the use of this knowledge in the design of coherent and efficient procedures for responding to epidemic situations.

The WHO is the principal global body responsible for collating, assimilating and implementing the results of research work and applying them to real-life epidemic situations. Many of the problems facing health officials are less scientific and more practical, such as non-reporting of cases, ignorance of the methods of transmission, the financial costs of developing, producing and distributing drug treatments, limited access to decent sanitation facilities, and difficulties in educating people about important facts of disease control; for example, proper burial procedures in order to aid eradication of the virus.

Policy makers are often forced to make quick and difficult decisions based upon the knowledge that they have at the time of an outbreak. Scientific rigour in research plays a vital role in influencing these decisions, by providing better understanding of the biological nature of infectious diseases and the dynamics of their spread. Well-funded research strategies are keys to increase our knowledge of infectious diseases; however, understanding the science is just one component of effective control, and one in which only collaborative effort between scientists and policy makers across many different fields can hope to achieve.

The meeting highlighted the scientific benefits that come from collaboration between mathematical and laboratory-based sciences in extending our understanding of both basic infectious disease processes as well as applied issues in disease control; such an approach will continue to provide the focus for research within CIDC.
